# Molecular characterization of echovirus 30-associated outbreak of aseptic meningitis in Guangdong in 2012

**DOI:** 10.1186/1743-422X-10-263

**Published:** 2013-08-23

**Authors:** Hong Xiao, Dawei Guan, Ruiyu Chen, Pingxi Chen, Corina Monagin, Wei Li, Juan Su, Cong Ma, Wanli Zhang, Changwen Ke

**Affiliations:** 1 Key Laboratory for Emergency Pathogen Detection, Guangdong Provincial Center for Disease Control and Prevention, Guangzhou 511430, Guangdong, China; 2 Luoding Center for Disease Control and Prevention, Luoding 527221, Guangdong, China; 3 Luoding People’s Hospital, Luoding 527221, Guangdong, China; 4 Metabiota, San Francisco, California 94104, USA

**Keywords:** Enterovirus, Aseptic meningitis, VP1 gene, Phylogenetic tree

## Abstract

**Background:**

Evaluation of the primary etiologic agents that cause aseptic meningitis outbreaks may provide valuable information regarding the prevention and management of aseptic meningitis. An outbreak of aseptic meningitis occurred from May to June, 2012, in Guangdong Province, China. In order to determine the etiologic agent, CSF specimens from 121 children hospitalized for aseptic meningitis at Luoding People’s Hospital of Guangdong Province were tested for virus isolation and identification.

**Results:**

Enterovirus RNA was positive in 62.0% of 121 CSF sspecimens by real-time polymerase chain reaction (RT-PCR). Amplification and sequencing of the VP1 region of enterovirus isolates revealed Echovirus 30 (E30) was the most common isolated serotype (80% of 40 enterovirus strains).For the molecular characterization of the E30 isolates, the VP1 gene sequence of 20 Luoding E30 isolates was compared pairwise using the MegAlign with reference strains from GenBank. The pairwise comparison of the nucleotide sequences of the VP1 genes demonstrated that the sequences of the strains differed from those of lineage groups C, D, E, F, and G. Reconstruction of the phylogenetic tree based on the VP1 nucleotide sequences resulted in a monophyletic tree, with seven clustered lineage groups. Most of the isolates were segregated from other lineage groups. Four E30 isolates causing this outbreak aggregated into the Lineage A cluster which was derived from E30 strains that circulated in other regions of China from 2003–2010.

**Conclusions:**

This study demonstrated the Luoding strains were a distinct lineage of E30, and a probable cause of this outbreak. The study also demonstrated that different E30 variants existed in the local meningitis outbreak.

## Background

Aseptic meningitis is a clinical syndrome characterized by meningeal inflammation that is not caused by any identifiable bacterial pathogen in the cerebrospinal fluid (CSF) [1]. Human enteroviruses (HEVs), small RNA viruses belonging to the Picornaviridae family, have been identified as the major etiologic agent of aseptic meningitis. Over 90% of viral meningitis cases are caused by HEVs [2]. HEVs can be transmitted by fecal-oral and respiratory routes, as well as by indirect contact via fomites and contaminated water. Echovirus 30 (E30), one of the distinct serotypes of HEVs, is a commonly isolated agent that causes sporadic to large outbreaks of aseptic meningitis in many regions of the world. In the last 15 years, multiple outbreaks and nationwide epidemics related to E30 have occurred worldwide [3-6]. In the U.S.A., E30 was a primary cause of meningitis outbreaks in 2003 and 2004 [4,7]. Moreover, several outbreaks of E30-associated meningitis have occurred in Asia in the last decade - Taiwan (2001), Korea (1997, 2008), Japan (2004 and 2006), and China (2003 and 2004) [3,5,6,8,9].

HEVs are non-enveloped viruses with a single positive stranded RNA genome of approximately 7.5 kb. Sixty-eight distinct serotypes of HEVs have been identified among more than 90 serotypes [4]. These viruses are genetically classified into four species: HEV-A, HEV-B, HEV-C, and HEV-D, E30 belongs to the B species (HEV-B), HEV-B is the largest specie comprised of 58 conventional serotypes, including Echovirus 1 ~ 7,9,11 ~ 21,24 ~ 27,29 ~ 33, coxsackie B1 ~ 6, coxsackie A9 and newly identified viruses (enterovirus 69, enterovirus 73 ~ 75, enterovirus 77 ~ 88, enterovirus 93, enterovirus 97 ~ 98, enterovirus 100 ~ 101, and enterovirus 106 ~ 107). Previous studies of the molecular epidemiology of E30 established four genotypes(designated 1 to 4) on the basis of VP1 sequences of 136 geographically dispersed E30 strains in 10 countries between 1956 and 1998 [7]. Other researchers have studied the molecular epidemiology of 112 European isolates of EV30 and presented a different classification into three genotypes (designated 1 to 3) and subdividing the last genotype into four new subgroups (3a to 3d) [10]. But interestingly, researchers found that after 1977, the two genotypes comprising the prototype strain Bastianni and the oldest European isolates circulating before 1976 had apparently disappeared. Furthermore, the oldest lineages of the prevailing genotype had likewise disappeared [10]. The genotype containing sequences after 1977 could be divided in sub-clusters. These sub-clusters showed a temporal relationship with each other. The currently circulating lineage displaced older ones in the late 1980s. Although the level of confidence of lineage clustering was lower, the temporal clustering resulting from the lineage clustering was very definite and many researchers use this classification to present the pattern of E30 evolution.

The capsids of HEVs are composed of four structural proteins (VP1, VP2, VP3, and VP4) and VP1-VP3 proteins are partially exposed on the capsid surface. The VP1 region, which is one of the main exposed regions of the viral capsids and the most variable region of the genome, provides the virus with distinct antigenic properties. In order to determine the genetic relationship between E30 isolates and any known enterovirus serotypes, the partial VP1 sequences have been compared with a database of complete enterovirus VP1 sequences of all E30 viruses. Additionally, phylogenetic analyses of sequence data of the VP1 region have been used extensively in molecular evaluations for epidemiology investigations [11,12]. In previous studies based on VP1 sequence analyses, E30 has evidenced a pattern of monophyletic evolution in which lineage displacement is correlated with the temporal dynamics of strains of this serotype [10,13]. E30 appears to circulate widely throughout Asia. Molecular characterizations are important to the epidemiology of E30; the determination of the VP1 sequences for isolated strains during an E30 meningitis outbreak may generate valuable information regarding enteroviral epidemiology. However, little information is currently available regarding the molecular characteristics of E30-associated aseptic meningitis in China. In this study, we reported an aseptic meningitis outbreak caused by E30, and evaluated the genetic diversity and molecular characteristics of the isolates via partial sequence analysis of the VP1 regions of the viral genome.

## Results

### The outbreak

Luoding City, between East longitude 111°03’08” to 111°52’44” and North latitude 22°25’11” to 22°57’34” is located in the central west region of Guangdong Province, with a modest subtropical monsoon climate and an annual average temperature of 22.10 degree Celsius.

**Figure 1:**
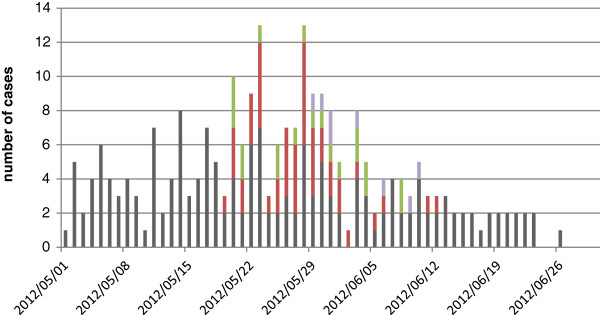
**Cases of Aseptic Meningitis in Luoding, Guangdong, in China, May 1**^**st **^**-June 30**^**th**^**, 2012.**

During May 2012, hospitalization for aseptic meningitis increased markedly at Luoding People's Hospital in Guangdong. An epidemiological curve of Aseptic Meningitis based on the surveillance data from May to June, 2012, is shown in Figure 1. The outbreak was characterized by two large peaks, which reached a maximum number of cases on May 23^rd^ and May 28^th^. A total of 246 patients were diagnosed clinically. Seventy-five cases were confirmed by laboratory analysis. The majority of patients (90%) was younger than 14 years old and had been admitted to the pediatric department. The sex ratio of male to female was 1.24:1 (136:110).

#### Enterovirus detection and molecular typing

Of 121 CSF samples, 75 (62.0%) were found positive for enterovirus RNA by real-time PCR. All 75 positive CSF samples were inoculated in RD and HEp2 cell lines for virus isolation; CPE was observed in 40 (53.3%).

All 40 isolates were typed molecularly. Sequencing and BLAST analysis identified strains as echovirus 30 (E30)(n = 32) and E6(n = 8). All the identified enterovirus isolates met the strain identification criteria with homologous serotypes including at least 75% nucleotide or 85% amino-acid homology between the enterovirus isolate and the reference strains in GenBank.

#### Sequence analysis of VP1 gene

**Figure 2:**
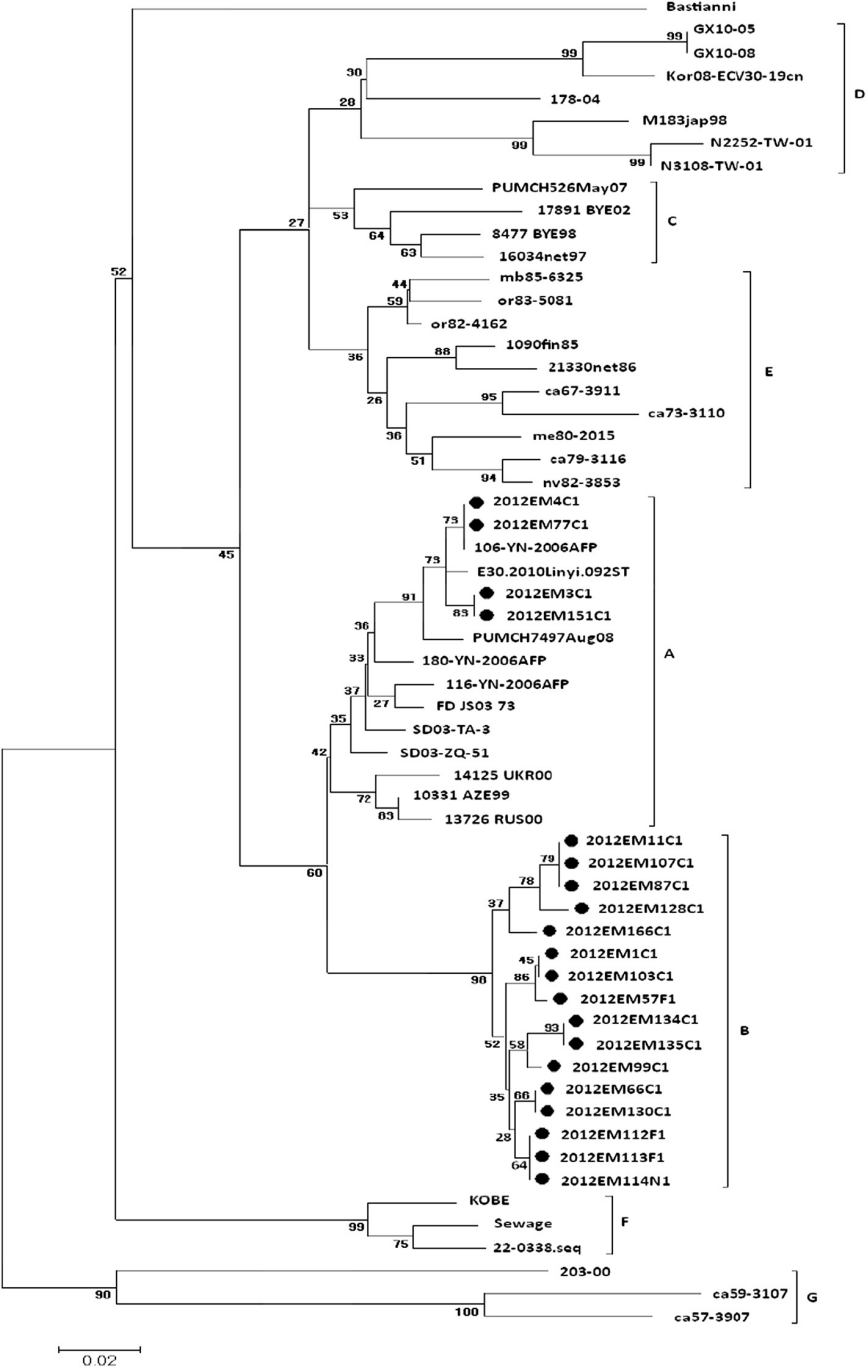
**Phylogenetic analysis of 2012 Luoding isolates and reference strains of E30.** Part nucleotide sequences of VP1 (nt 2,627-2,951) were analyzed via the neighbor-joining methods and the tree was constructed using the MEGA (version 4.0, software package). The nucleotide position is relative to the sequence of Bastianni strain of E30 prototype; Luoding E30 strains are marked using the symbol; The bracketed regions indicate Lineage, marked with **A**, **B**, **C**, **D**, **E**, **F**, **G**.

In order to analyze the genetic characteristics of E30, the sequences of 20 E30 strains isolated from aseptic meningitis patients were selected (patient information listed in Table 1). The VP1 sequences of 20 isolates were determined and compared with 39 reference strains from GenBank database. The identity of the nucleotide sequence for the VP1 region within these isolates was high (> 90%) in the 324 bp. The 16 nucleotide sequences of 2012EM isolates differed from the Group A lineage of E30 isolated in China between 2003 ~ 2010. Pairwise comparison of the nucleotide sequences of the VP1 genes demonstrated that the sequences of the strains differed from those of the isolates of lineage groups C, D, E, F and G (Figure 2). Nucleotide sequences encoding for VP1 (nt 2,627-2,951) were analyzed via the neighbor-joining methods and trees were constructed using the MEGA software package, version 4.0. The reconstruction of the phylogenetic tree based on the VP1 nucleotide sequences of the isolates and the E30 reference strains supported a monophyletic VP1 tree. According to the clusters formed via phylogenetic analysis, E30 was clustered into seven lineage groups. Sixteen isolates were segregated from the other previously reported lineage groups, thus suggesting that the strains were a distinct lineage of E30, and may plausibly have been the major cause of this outbreak.

**Table 1:** E30 isolate used for phylogenetic analysis

**Isolation**	**Sampling date**	**Specimen**	**Patient age**	**Clinical**
2012EM1C1	20-May-2012	CSF	8	Headache, Fever, Vomiting
2012EM3C1	20-May-2012	CSF	7	Headache, Fever, Vomiting
2012EM4C1	20-May-2012	CSF	8	Headache, Fever, Vomiting
2012EM11C1	21-May-2012	CSF	7	Headache, Fever, Vomiting
2012EM57C1	21-May-2012	CSF	9	Headache, Fever, Vomiting
2012EM66C1	23-May-2012	CSF	10	Headache
2012EM77C1	25-May-2012	CSF	12	Headache
2012EM87C1	25-May-2012	CSF	12	Headache
2012EM99C1	27-May-2012	CSF	11	Headache
2012EM103C1	28-May −2012	CSF	9	Headache
2012EM107C1	29-May −2012	CSF	8	Headache
2012EM112F1	30-May −2012	CSF	8	Headache, Fever
2012EM113F1	31-May −2012	CSF	5	Fever, Vomiting
2012EM114N1	1-Jun-2012	CSF	4	Fever, Vomiting
2012EM128C1	3-Jun-2012	CSF	2	Fever, Vomiting
2012EM130C1	3-Jun-2012	CSF	2	Fever, Vomiting
2012EM134C1	4-Jun-2012	CSF	7	Headache, Fever
2012EM135C1	4-Jun-2012	CSF	5	Fever
2012EM151C1	8-Jun-2012	CSF	9	Fever
2012EM166C1	8-July-2012	CSF	7	Fever

#### Analysis of VP1 amino acid sequence

**Figure 3:**
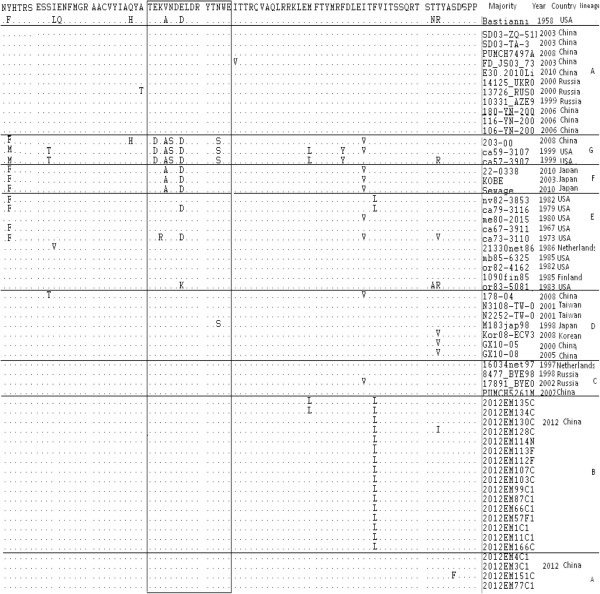
Comparison of the deduced amino acid sequence of 59 E30 strains in the of VP1 sequence.

The BC loop within the VP1 region was identified as crucial for the reactivity of type-specific antibodies. Almost all of the isolates harbored substituted amino acids within VP1 BC loop region (Figure 3). On the amino acid sequence comparison of VP1, sixteen Luoding strains of the Lineage B were substituted L for F at the 122 amino acid position. The 2012EM128C1 isolate was substituted at the 133 amino acid position (T133I), the 2012EM151C1 isolate was substituted at the 136 amino acid position (S136F), and two Luoding strains of the Lineage B were substituted L for M at the 110 amino acid position (Figure 3).

## Discussion and conclusion

Echoviruses were initially isolated from stools samples of asymptomatic individuals in 1951 and many researchers have asserted that echoviruses are associated with a variety of human diseases, including asymptomatic infections, febrile illness, aseptic meningitis, and severe diseases in newborns. E30, a member of the HEV-Bs, has caused a number of outbreaks of aseptic meningitis in many different countries. Several outbreaks of E30-associated meningitis have been reported in Asia over the past decade [2,3,5,6,9]. E30 is receiving increasing attention in China as well. Zhao et al. reported an outbreak of aseptic meningitis associated with E30 in Jiangsu Province in 2003 [6]. E30 was also found to be involved in several outbreaks of aseptic meningitis and hand-foot-and-mouth disease in the neighboring provinces (Zhejiang and Shandong) [8]. E30 appears to be widely circulating in these regions and understanding its pathogenesis and molecular epidemiology will have important public health implications. In this study, we observed that, during the period from May to June of 2012, hospitalized patients with aseptic meningitis increased at the Luoding People’s Hospital. In our etiologic analysis, E30 was the principal isolate from 121 children hospitalized for aseptic meningitis, which suggests that E30 was in fact the main etiologic agent of this outbreak.

Many previous studies have demonstrated the genetic diversity among strains of the E30 serotype [6,13]. Molecular investigations based on VP1 sequence analysis demonstrated that the genetic diversity of E30 has been characterized by sequential displacements among multiple genetic variants [4]. Point mutations of E30, which have been associated with meningitis outbreaks, involve substantial genetic diversity in the gene encoding for the VP1 polypeptide [5,14,15]. Whereas previous molecular epidemiological investigations have suggested that different E30 variants can exist among circulating strains in local meningitis outbreaks and that a given variant can be associated with several outbreaks in distant geographical areas, each outbreak was generally associated with only one or a few viral variants [10]. Recent phylogeography investigations of human E30 have demonstrated that viral lineages were closely related to the time period in which they were isolated [4,6,13]. In the United States, Canada and Europe, some researchers found that the genotype composed of the two oldest isolates (1975–1976) seems to have disappeared and representatives of the prototype Bastianni cluster were not seen. The genotype containing sequences after 1977 could be divided in subclusters or lineages. To study of the molecular epidemiology of E30 in China, a total of 59 E30 VP1 sequences were analyzed, including prototype Bastianni strain, 5 Russian sequences (1998–2002), 4 Japanese sequences(1998–2010), 2 Netherland sequences (1986,1997), 1 Finland sequence (1985), 10 Amercian sequences (1967–1999), 2 Taiwan sequences (2001), 1 Korean sequence (2008) and 13 Chinese previous reported sequences(2000–2010). The 13 Chinese strains were isolated from five different regions (Zhejiang, Jiangsu, Yunnan, Shandong and Guangxi). The reconstructed VP1 tree showed that the majority of E30 isolates causing this outbreak aggregated into the tight cluster of Lineage B, which was different from E30 strains that circulated in other countries in 1958–2008 and in China from 2000–2010. Only four E30 isolates causing this outbreak aggregated into the Lineage A. But all these E30 isolates causing this outbreak weren’t belonging to the two oldest isolates genotype (Bastianni and M183jap98).

The virus-encoded RNA-dependent RNA-polymerase shows a high error frequency, which is due to the lack of a proof-reading mechanism. As a result, viruses exit as mixtures of genetic variants or quasispecies. In addition, homologous intertypic and intratypic recombination occurs frequently between RNA strands of the same or different enterovirus serotype. As a result, numerous genetic lineages, so called genotypes, of any given enterovirus serotypes circulate worldwide [10,13]. Recombination frequency was tightly correlated with VP1 divergence, the VP1 phylogenies represent a much better approximation of the evolutionary history of the E30 serotype than other regions and allow time of origin and differentiation of structural gene regions to be more easily inferred [16].The genetic diversity of E30 provided us with different classifications of the genetic variants of E30. Our phylogeographic investigations of human E30 have demonstrated that seven viral lineages, A-G, were closely related to the time period in which they were isolated and the temporal clustering resulting from the lineage clustering was very definite. The A lineage E30, which was initially isolated in the Community of Independent States in 1999, caused aseptic meningitis outbreaks in the Chinese provinces of Zhejiang, Yunnan and Shandong from 2003–2010. The D lineage E30 circulated throughout Japan in1998, Taiwan in 2000–2001, Korea in 2008 and Guangxi Province of China in 2000 and 2005. E30 does not seem to be geographically restricted to certain regions, as a given lineage circulates in different regions of the world simultaneously.

Owing to the paucity of information regarding the VP1 sequence of E30 isolated in China before 2000, we were only able to assess genetic diversity among E30 strains isolated in China from 2000 to 2010. Comparisons of VP1 amino acid sequences also demonstrate that a distinct lineage B of most of the E30 strains isolated from Guangdong in 2012 differs from the E30 strains isolated previously in Asia. Only a few of the strains (4 strains) identified with the E30 strains isolated previously in China. Some researches considered E30 differs from other enteroviruses. The molecular epidemiology of E30 may relate to influenza virus epidemiology, where prevailing lineages displace the less established lineages on the basis of immune escape, and a single lineage at a time appears to circulate around the world. A lineage displacement could be considered to be antigenic drift in E30 epidemiology [6,9,13]. The BC loop within the VP1 region was identified as crucial for the reactivity of type-specific antibodies [17]. In our study all of the Luoding isolates harbored substituted amino acids within the BC loop region, but sixteen Luoding isolates of the Lineage B appeared to have substitution at 122 (F122L). This substitution was suggested as a reason of occurrence of lineage displacement and the antigenic drift.

In conclusion, the present report demonstrated that the Luoding strains were a distinct lineage B of E30, and a probable cause of this outbreak. Different E30 variants and other serotype echoviruses co-circulated in the same area and caused meningitis. It is necessary to establish an enterovirus molecular surveillance system in China in order to gain a better understanding of viral transmission and evolution.

## Materials and methods

### Clinical samples and patients

An epidemic of encephalitis in Luoding which is an economically less developed areas of Guangdong Province was reported by Guangdong Provincial Center for Disease Control and Prevention (GDCDC) from May to June 2012.

121 cerebrospinal fluid (CSF) specimens were collected from children (age ≤ 14 years) with symptoms of encephalitis, were collected at the time of admission to Luoding People’s Hospital. The CSF specimens were immediately transported and stored with proper cold chain storage in the laboratory, and stored at −70°C until further use. A case of encephalitis was defined as fever with altered sensorium lasting more than 24 h with ≥1 of the following symptoms: fever, seizures and CSF pleocytosis, with neuroimaging finding indicating parenchymal involvement. The dominant clinical manifestation of this epidemic was high-grade fever, altered sensorium, convulsions and meningeal signs. For the use of these CSF samples, written informed consents from all participants (their parents or legal guardians) involved in survey were obtained. The study was approved by the ethics committee of the Guangdong Provincial Center for Disease Control and Prevention, and was in compliance with the Helsinki Declaration.

### Enterovirus detection by one-step Real-time PCR

RNA was extracted from CSF with QiaAmp viral RNA kit (Qiagen, Hilden, Germany). In summary, 200 μl of prepared CSF or virus culture were treated with 200 μl of guanidine hydrochloride extraction buffer containing 28 μg/ml of carrier RNA, followed by alcohol precipitation. The precipitates were applied to the QIAamp MinElute column, and the viral nucleic acid was absorbed onto the silica gel membrane. The pellet was then re-suspended in 50 μl of RNAase-free water.

Enterovirus detection was processed in the one step real-time PCR, Real time PCR was conducted in an ABI7500 Fast Real-Time PCR System (Applied Biosystems Inc., USA) using a Diagnostic kit for Enterovirus RNA Real-time reagent (bioPerfectus technologies, China). According to the instructions, Fluorescence signals were measured every cycle at the end of the annealing step. A real-time PCR amplification plot of typical “S” and Ct ≤ 35.5 indicated that the sample was positive for enterovirus, otherwise the amplification plots were not typical “S” or Ct > 35.5 indicating that the sample was negative.

### Virus isolation and molecular typing

Virus isolation from the positive samples was performed in human rhabdomyosarcoma cells (RD) and human epithelium larynx (HEp 2) cell lines. When complete cytopathic effect (CPE) was observed, culture supernatants from positive samples were collected and stored at −70°C until use.

Molecular typing of enterovirus isolates was performed by semi-nested real-time reverse-transcriptase-polymerase chain reaction (RT-PCR) amplification of the VP1 region of the virus genome using the AN88 and AN89 primers as described earlier [18]. The amplicons were separated by 2% agarose gel electrophoresis, purified with the QIAquick PCR Purification Kit (QIAGEN, Hilden, Germany), and sequenced on the ABI Prism automated sequencer by 3100 Genetic Analyzer (Applied Biosystems) with the same primers (AN88 and AN89).

### Phylogenetic analysis

Molecular identification of each isolate was done by pairwise comparison of the VP1 amplicon sequence with a database of all EV serotypes using the BLAST program (http://www.ncbi.nlm.gov/BLAST) from GenBank. Strains that had the highest nucleotide similarity (at least 75% identical in VP1 sequence) belong to the same serotype [19]. Multiple sequence alignments with the respective reference strain sequences were generated with the Clustal W program (http://www.ebi.ac.uk/clustalw). A phylogenetic tree was computed using the neighbor-joining method with bootstrap 1000 replicates in MEGA 4.0 software (http://www.megasoftware.net).

## Abbreviations

HEVs: Human enteroviruses; E30: Echovirus 30; CSF: Cerebrospinal fluid; RT-PCR: Real-time reverse transcriptase PCR; RD: Human rhabdomyosarcoma cells; HEp 2: Human epithelium larynx; CPE: Cytopathic effect.

## Competing interests

We confirm that the manuscript has been read and approved by all named authors and that there are no other persons who satisfied the criteria for authorship but are not listed. We further confirm that the order of authors listed in the manuscript has been approved by all of us. We further confirm that any aspect of the work covered in this manuscript that has involved human patients has been conducted with the ethical approval of all relevant bodies and that such approvals are acknowledged within the manuscript.

## Authors’ contributions

HX performed the experiment, data analysis and prepared the manuscript; CK designed the study and prepared the manuscript; DG data analysis and performed the real-time PCR experiments; WL, JS, CM and WZ performed the virus isolate experiment; RC and PC coordinated and provided the collection of patient materials; CM revised the manuscript. All authors read and approved the final manuscript.
